# Correlation Between Athlete Status and Clinical Features of Polycystic Ovary Syndrome in Collegiate Females

**DOI:** 10.7759/cureus.88073

**Published:** 2025-07-16

**Authors:** Lauren Hibshman, Madeleine Schlehr, Catelyn Rueger, Nathan Quint, Diana Speelman

**Affiliations:** 1 Sports Medicine, Penn Medicine, Philadelphia, USA; 2 Medicine, Lake Erie College of Osteopathic Medicine (LECOM), Erie, USA; 3 Psychiatry, Lake Erie College of Osteopathic Medicine (LECOM), Erie, USA; 4 Pediatrics, Wareham Pediatrics, Boston, USA; 5 Statistics, Lake Erie College of Osteopathic Medicine (LECOM), Erie, USA; 6 Biochemistry, Lake Erie College of Osteopathic Medicine (LECOM), Erie, USA

**Keywords:** athlete, collegiate athlete, hyperandrogenism, menstrual irregularity, pcos, physical activity

## Abstract

Background

This study examined the correlation between athlete status and clinical features of polycystic ovarian syndrome (PCOS) in collegiate females between the ages of 18 and 22.

Methods

This was an anonymous, online survey-based correlational study. Survey invitations were emailed to athletic directors and science department chairpersons at colleges and universities in New York, Pennsylvania, Ohio, and New Jersey for distribution to students. Survey items asked about respondents’ general medical history, clinical signs associated with PCOS, physical activity, and diet. Correlations were analyzed using chi-square tests.

Results

Collegiate female athletes (n = 164) were less likely than non-athletes (n = 278) to report hyperandrogenism (2 (1.2%) vs. 15 (5.4%), p = 0.02742), menstrual irregularity (24 (14.6%) vs. 65 (23.4%), p = 0.02674), anti-androgen medication use (0 (0%) vs. 11 (4.0%), p = 0.008496), or PCOS diagnosis (5 (3.0%) vs. 26 (9.4%), p = 0.01218). No significant correlation was found with athlete status and acne, hirsutism, or special diet. Positive correlations of BMI with menstrual irregularity and PCOS diagnosis were consistent with previous studies. No significant correlation was found with BMI and acne, hyperandrogenism, or anti-androgen medication use.

Conclusions

There was an association between being a collegiate athlete and reduced prevalence of PCOS diagnosis, hyperandrogenism, and menstrual irregularity. Further studies are needed to determine if physical activity, at the level of a collegiate athlete, may help reduce the clinical expression of PCOS diagnostic features.

## Introduction

Polycystic ovary syndrome (PCOS) is a very common endocrine disorder in women of reproductive age, affecting 5-18% of this population [1]. Diagnosis is made in the presence of at least two of three criteria: hyperandrogenism, menstrual irregularity secondary to oligo-ovulation, and polycystic ovaries [2]. Exclusion of other hormonal disorders that could cause hyperandrogenism or menstrual irregularity is also warranted. In addition to the diagnostic criteria and reproductive effects, women with PCOS are more likely to have metabolic dysfunction, which includes obesity, insulin resistance, type 2 diabetes, and nonalcoholic fatty liver disease [3]. Diagnosis may occur as early as adolescence but is often not made until later [4].

Women with PCOS are also reported to have some differences in muscle composition compared to women without the disorder. Increased lean mass [5,6], greater muscle strength [7], and enhanced muscle strength after resistance training [8] have been reported in women with PCOS. The elevated androgens present in women with PCOS may contribute to the greater muscle size [7,9], although this correlation is not always found [5]. Another possible explanation for greater muscle mass and strength could be the elevated levels of insulin in women with PCOS, as this is a growth factor [5].

Increased muscle mass and strength could be a natural advantage for young female athletes with PCOS. To determine if there is a correlation between signs and symptoms of PCOS in young women who participate in organized sports, we used a cross-sectional study design to survey a sample of females between the ages of 18-22 who were enrolled in a college or university in the northeastern United States. We hypothesized that a greater percentage of female athletes would report a PCOS diagnosis or signs and symptoms consistent with PCOS compared to non-athletes at the same schools.

## Materials and methods

This study was reviewed by the Lake Erie College of Osteopathic Medicine Institutional Review Board (Protocol 25-136) and determined to be exempt from the requirement for IRB approval. The aim of this study was to collect anonymous survey data from female college students to examine the correlation between athlete status (athlete or non-athlete) and clinical features of PCOS.

Respondent recruitment

Participants were recruited via email invitation, originating from the investigators and sent out by Athletic Directors and Science Department Chairpersons at colleges and universities in New York, Pennsylvania, Ohio, and New Jersey within the United States. The email inviting individuals to participate indicated that the survey was conducted by medical students researching the hormonal health of female college students and that all responses would be anonymous. Females who chose to participate were able to anonymously complete a one-time survey using Google Forms. Participant surveys were collected between May 10, 2018, and May 16, 2020.

Respondent inclusion and exclusion

Inclusion criteria were female participants between the ages of 18 and 22 and enrolled in a college or university in one of the states indicated above. Exclusion criteria were younger than 18 or older than 22 years of age, self-reported thyroid disorder, self-reported Cushing syndrome or disease, or non-response to one or more survey items for the primary measures. A total of 442 responses meeting these criteria were used for data analysis.

Study design and survey

This was a survey-based correlational study. Survey respondents answered questions about general medical history (including age, height, weight, acne or acne treatment, hirsutism, medications, diet, other hormone disorders), family medical history (obesity, type 2 diabetes, metabolic syndrome), menstrual history, physical activity and participation in team sports, and familiarity with PCOS. Survey items are shown in Table 1. Respondents who answered “yes” to the question about participation in an NCAA (National Collegiate Athletic Association), NAIA (National Association of Intercollegiate Athletics), varsity, club, or recreational sports team were classified as athletes; all others were classified as non-athletes. The primary outcome measures were the correlations between being an athlete and indications of acne (items 5 and 6), hirsutism (item 7), both acne and hirsutism, or diagnosis of hyperandrogenism (item 10); irregular menstrual cycle length or frequency (items 17 and 18); or diagnosis of PCOS (item 31).

**Table 1:** Survey items.

Survey Items
1. What school do you attend? Drop-down menu
2. How old are you? Open response
3. Height (feet and inches) and 4. Weight (pounds) Open responses
5. Have you ever been treated by a physician for acne? Y/N
6. Do you use any over-the-counter medications for acne? Y/N
7. Do you have unwanted or excess hair growth on any of the following? Select all that apply: face (upper lip, chin, jawline), chest, thighs, none
8. Select any of the following prescribed medications that you take. Select all that apply: hormones (e.g., androgens, cortisol, steroids, prednisone, estrogen, progesterone), contraceptives, anti-androgen, insulin, insulin sensitizer, acne medication, none
9. Has a physician ever diagnosed you with any of the following thyroid disorders? Select all that apply: hyperthyroidism, hypothyroidism, none
10. Has a physician ever diagnosed you with hyperandrogenism (high testosterone or androgen levels)? Y/N
11. Has a physician ever diagnosed you with Cushing’s syndrome (an adrenal gland disorder)? Y/N
12. Have you noticed any weight gain over the past four years that you cannot attribute to normal growth or that seems excessive based on your food intake? Y/N
13. Do you follow a special diet? If so, what is it? Open response
14. Family History: Is anyone in your immediate family considered overweight or obese? Y/N
15. Family History: Has a physician diagnosed your biological mother or father with Type 2 diabetes? Select all that apply: mother, father, unsure, neither
16. Age of your first menstrual period? Open response
17. On average, how many days between day 1 of your period and day 1 of your next period? (Day 1 = first day of bleeding) Open response
18. How many periods have you had in the last 12 months? Open response
19. Do you track your menstrual cycle (e.g., by marking it on a calendar)? Y/N
20. Are you on an NCAA, NAIA, or varsity team, a club team, or a recreational team? Y/N
21. Answered N to item 20: How many hours do you participate in aerobic exercise each week (e.g., walking, running, cycling, swimming, or similar activity)? Open response
22. Answered N to item 20: How many hours do you participate in anaerobic exercise each week (e.g., weight-lifting, interval training, yoga, pilates)? Open response
23. Answered Y to item 20: What sport(s) do you play? Select all that apply: basketball, crew, cross country, equestrian, fencing, field hockey, golf, gymnastics, ice hockey, lacrosse, soccer, softball, squash, swimming & diving, tennis, track & field, volleyball, other (write-in)
24. Answered Y to item 20: How many hours per week do you practice in-season? Open response
25. Answered Y to item 20: How many hours per week do you weight-train in-season? Open response
26. Answered Y to item 20: How many hours per week do you practice off-season? Open response
27. Answered Y to item 20: How many hours per week do you weight-train off-season? Open response
28. Answered Y to item 20: Is your menstrual cycle during your sports season different than during your off season? Y/N
29. Answered Y to item 20: If you answered yes to the previous question, please describe the changes you have noticed. Open response
30. Are you familiar with polycystic ovary syndrome (PCOS)? Y/N
31. Has a physician ever diagnosed you with PCOS? Y/N
32. Would you be interested in learning more about PCOS? Y/N *A yes response took participants to an information page about PCOS that was published by the National Institutes of Health: https://www.nichd.nih.gov/health/topics/pcos/conditioninfo

Secondary outcomes measured were the correlation between being an athlete and the use of anti-androgen medication (item 8), unexplained weight gain (item 12), and following a specific diet (item 13). In addition, secondary outcome measures included the correlation between body mass index (BMI) categories and acne, hirsutism, both acne and hirsutism, hyperandrogenism diagnosis, anti-androgen medication, irregular menstrual cycle length or frequency, and diagnosis of PCOS. BMI was calculated from respondents’ height and weight using the formula: (weight (in kilograms)) / (height (in meters))2. Categories for BMI are based on those given by the Centers for Disease Control and Prevention (CDC): < 18.5 underweight, 18.5-24.9 healthy weight, 25-29.9 overweight, 30-39.9 class 1 and 2 obesity, and > 40 class 3 (severe) obesity [10]. The correlation between NCAA, NAIA, or varsity sport participation, specifically, and hyperandrogenism or activity-dependent changes in cycle regularity was also investigated.

Data collection

Anonymous responses were collected in Google Forms; no email addresses were collected. Dichotomous data were coded as 0 or 1 for statistical analysis. 

Statistical analysis

Data were analyzed using the R program. Prior to statistical analysis, data were checked for normality using the D’Agostino-Pearson test. Comparison of demographic data between athletes and non-athletes was made using Mann-Whitney U tests. For the correlations with data approximating a standard distribution, the chi-square test was used. When the data were not normally distributed, the chi-square test following the Monte Carlo simulation was used. Significance was set at p < 0.05. Effect sizes were calculated using Cramer’s V. The effect size was determined to be small, medium, or large based on the degrees of freedom. For df = 1, this corresponded to effect sizes of 0.10, 0.30, and 0.50. For df = 4, this corresponded to effect sizes of 0.05, 0.15, and 0.25.

## Results

A total of 13 schools were represented, with 10 schools including responses from multiple participants. After applying inclusion and exclusion criteria, a total of 442 respondents were included in the data analyses from 13 schools. Female college students who indicated that they were members of NCAA, NAIA, varsity, club, or recreational team were coded as athletes (n = 164); all others were coded as non-athletes (n = 278). Athletic activities reported included field hockey (37, 22.6%), soccer (28, 17.1%), swimming and diving (24, 14.6%), basketball (15, 9.1%), softball (12, 7.3%), cross country (11, 6.7%), crew (9, 5.5%), track and field (9, 5.5%), volleyball (8, 4.9%), cheerleading (7, 4.3%), lacrosse (7, 4.3%), tennis or squash (4, 2.4%), dance team (3, 1.8%), ultimate frisbee (3, 1.8%), archery (2, 1.2%), rugby (1, 0.6%), equestrian (1, 0.6%), and golf (1, 0.6%). Some respondents reported more than one athletic activity. Demographics of respondents are shown in Table 2, with values for continuous variables expressed as means with standard deviation. Respondents classified as athletes had a lower average BMI (24.1 vs. 26.9, p = 0.0001), averaged more cycles per year (10.8 vs. 9.6, p = 0.0039), reported more hours of aerobic exercise in-season and off-season (13.6 and 8.3 vs. 3.1, p < 0.0001 for each), and more hours of anaerobic exercise in-season and off-season (3.0 and 4.2 vs. 1.3, p < 0.0001 for each) than non-athletes.

**Table 2:** Demographics of respondents. * p < 0.05

Variables	Athletes (n = 164)	Non-athletes (n = 278)	P value
Age (years)	19.7 + 1.2 *	20.1 + 1.3	0.0033
BMI (calculated from height & weight)	24.1 + 3.6 *	26.9 + 6.5	0.0001
Average # of cycles per year	10.8 + 3.5 *	9.6 + 4.2	0.0039
Tracks menstrual cycle (% yes)	58%	55%	
Aerobic exercise (hours/week), in-season	13.6 + 6.1 *	3.1 + 2.8	< 0.0001
Aerobic exercise (hours/week), off-season	8.3 + 6.0 *	3.1 + 2.8	< 0.0001
Anaerobic exercise (hours/week), in-season	3.0 + 3.3 *	1.3 + 1.7	< 0.0001
Anaerobic exercise (hours/week), off-season	4.2 + 3.6 *	1.3 + 1.7	< 0.0001

Correlations between athlete status and clinical features of PCOS

There was no correlation between being an athlete and having acne (78 (47.6%) athletes vs. 132 (47.5%) non-athletes; χ2(1, 442) = 0.00026, p = 0.9872) or hirsutism (106 (64.6%) athletes vs. 184 (66.2%) non-athletes; χ2(1, 442) = 0.11024, p = 0.7399). There was no statistically significant difference between the percentage of athletes and non-athletes who reported both acne and hirsutism (106 (64.6%) athletes vs. 184 (66.2%) non-athletes; χ2(1, 442) = 0.11024, p = 0.7399), which is suggestive of hyperandrogenism. There was, however, a correlation between being an athlete and reported hyperandrogenism, with 5.4% (n = 15) of non-athletes reporting the diagnosis of hyperandrogenism compared to only 1.2% (n = 2) of athletes (χ2(1, 442) = 4.8644, p = 0.02742, Cramer’s V = 0.1049). There was also a correlation between athlete status and use of an anti-androgen, with a greater percentage of non-athletes reporting use of this type of medication (11 (4.0%) vs. 0 (0%); χ2(1, 442) = 6.6548, p = 0.008496, Cramer’s V = 0.1227).

Compared to non-athletes, female athletes were less likely to have fewer than eight menstrual cycles per year (24 (14.6%) vs. 65 (23.4%); χ2(1, 442) = 4.9077, p = 0.02674, Cramer’s V = 0.1054), although there was no correlation between athlete status and cycles greater than 35 days in length (35 (21.3%) athletes vs. 57 (21.5%) non-athletes; χ2(1, 442) = 0.043934, p = 0.834). A lower percentage of athletes reported a PCOS diagnosis (5 (3.0%) vs. 26 (9.4%); χ2(1, 442) = 6.285, p = 0.01218, Cramer’s V = 0.1192).

Correlations between body mass index and clinical features of PCOS

There was a correlation between BMI category and having fewer than eight menstrual cycles per year (χ2(4, 442) = 13.15, p = 0.009495, Cramer’s V = 0.1725). Respondents with a BMI greater than 29.9 were more likely to report fewer than eight cycles per year: 0% underweight (0 of 7), 17.6% healthy weight (40 of 227), 16.8% overweight (21 of 125), 34.3% class 1 and 2 obesity (24 of 70), and 30.8% severe obesity (4 of 13). A positive correlation was found between BMI and PCOS diagnosis (χ2(4, 442) = 22.07, p = 0.001999, Cramer’s V = 0.2234). By BMI category, the percentage of respondents with a PCOS diagnosis were 0% (0 of 7) underweight, 3.1% (7 of 227) healthy weight, 7.2% (9 of 125) overweight, 17.1% (12 of 70) class 1 and 2 obesity, and 23.1% (3 of 13) severe obesity. Respondents with a BMI less than 18.5 were less likely to report hirsutism (χ2(4, 442) = 9.2883, p = 0.04448, Cramer’s V = 0.145). By BMI category, the percentage of respondents reporting hirsutism were 14% (1 of 7) underweight, 68% (155 of 227) healthy weight, 63% (79 of 125) overweight, 66% (46 of 70) class 1 and 2 obesity, and 69% (9 of 13) severe obesity.

Though it did not reach statistical significance, there was a trend toward positive correlation between BMI and the presence of both acne and hirsutism (χ2(4, 442) = 9.2883, p = 0.06097, Cramer’s V = 0.145). There was no significant correlation between BMI and acne (χ2(4, 442) = 7.7544, p = 0.08846), anti-androgen medication use (χ2(4, 442) = 3.995, p = 0.3973), hyperandrogenism (χ2(4, 442) = 8.0465, p = 0.08996), or cycle length greater than 35 days (χ2(4, 442) = 3.6381, p = 0.4698). There was a positive correlation in respondents who reported unexpected weight gain (χ2(4, 442) = 113.42, p = 0.0004998, Cramer’s V = 0.5066) with increasing BMI: 0% (0 of 7) underweight, 10.6% (24 of 227) healthy weight, 28.0% (35 of 125) overweight, 61.4% (43 of 70) class 1 and 2 obesity, and 100% (13 of 13) severe obesity.

Additional correlations

Female students who played on NCAA, NAIA, or varsity teams (n = 146) were more likely than non-varsity students (n = 296) to report changes in menstrual cycle regularity as their physical activity levels increased during the year (63 (43.2%) vs. 65 (22.0%); χ2(1, 442) = 21.342, p < 0.0001, Cramer’s V = 0.2197). Changes reported during the in-season for athletes included increased cycle length or more time between periods (22 specified this change) and lighter flow (17 specified this change). Varsity athletes also trended toward being less likely to report hyperandrogenism than non-varsity students (2 (1.4%) vs. 15 (5.1%); χ2(1, 442) = 3.6149, p = 0.05726, Cramer’s V = 0.09043).

Respondents categorized as athletes were no more likely to report a special diet than non-athletes (20 (12.2%) of 164 vs. 41 (14.7%) of 278, respectively; χ2(1, 442) = 0.56518, p = 0.4522). Of the special diets reported by athletes and non-athletes, respectively, these included caloric restriction or intermittent fasting (n = 4 (20%), 11 (27%)), gluten-free (n = 5 (25%), 6 (15%)), dairy-free (n = 4 (20%), 4 (10%)), vegetarian or vegan (n = 4 (20%), 1 (2%)), low-carbohydrate or keto (n = 1 (5%), 5 (12%)), low-fat (n = 1 (5%), 2 (5%)), pescatarian (n = 2 (10%), 0 (0%)), and high protein (n = 0 (0%), 1 (2%)). Some respondents reported more than one type of special diet.

## Discussion

In this cross-sectional survey of collegiate women 18-22 years old, female athletes were less likely to report signs and symptoms consistent with PCOS. Significantly lower percentages of the athletes had hyperandrogenism, anti-androgen medication use, menstrual irregularity, and PCOS diagnosis; the effect size for each of these was small. No significant differences were observed in the percentages of athletes and non-athletes reporting acne, hirsutism, or acne and hirsutism together. Athletes had a lower mean BMI than non-athletes and reported more hours of both aerobic and anaerobic exercise during both their sport season and off-season. Women with a BMI of 30 or above were more likely than women with a lower BMI to report menstrual irregularity or a PCOS diagnosis; the effect sizes were medium and large, respectively. Together, these findings suggest that the prevalence of PCOS in young, collegiate female athletes may be lower than in non-athletes in the same age group who attend the same schools.

Contrary to our hypothesis, young athletes were less likely to report features associated with PCOS. There are a few possible ways to interpret this, which are not mutually exclusive. Regular physical activity can help with weight management, as well as the mitigation of the clinical signs of hyperandrogenism and menstrual irregularity. Indeed, a healthy diet and regular exercise are first-line lifestyle recommendations for women with PCOS, with demonstrated improvement in menstrual regularity and androgen levels [11,12]. The athletes in this study had a lower average BMI and reported more hours of physical activity than the non-athletes, which may partially explain the difference in the clinical features and diagnosis of PCOS. Females who have been physically active throughout adolescence and into young adulthood may also be less likely to exhibit unexpected weight gain or have metabolic dysfunction that is often found in women with PCOS [13-16]. Furthermore, exercise is known to moderate insulin levels and sensitivity [17], and the pathophysiology of PCOS can be exacerbated by hyperinsulinemia [18-20]; therefore, regular physical activity may delay or prevent the expression of PCOS in women who are susceptible to it. Women with the metabolic features of PCOS may also be less likely to participate in physical activity [21,22].

Our findings support the greater likelihood of a PCOS diagnosis with increasing BMI [23]. This may indicate a greater risk of PCOS development with increasing adiposity in childhood, adolescence, or adulthood [24]. There may also be a greater likelihood of receiving a PCOS diagnosis with higher BMI due to provider bias [25]. Women surveyed were more likely to report menstrual irregularity (< 8 cycles per year) with increasing BMI, which is consistent with disruption of reproductive function when obesity is present [1,23,24]. Reports of unexpected weight gain also increased with increasing BMI, which could be related to a number of physiological, psychological, and environmental factors [3,26-28].

A secondary goal of our study was to assess awareness about PCOS in this age group. Diagnosis of PCOS is often delayed beyond the onset of signs and symptoms [4,25], which can result in protracted implementation of helpful lifestyle modifications or medications. Of our respondents, 53.4% indicated that they were not familiar with PCOS, although some of these women reported clinical features consistent with the disorder. We asked about cycle length and the number of menstrual periods per year and found the latter to provide more reliable responses. From the clinical and research perspectives, it is critical to ask about both cycle length and cycles per year, since some respondents will misinterpret cycle length as menstrual period length; the ability to accurately identify menstrual irregularity is often crucial for the diagnosis [2,20]. It is also important to educate female adolescents and young adults about PCOS, since this disorder can impact reproductive, metabolic, cardiovascular, and psychological health across her lifespan [3,29]. Patient education and earlier diagnosis are also important for implementing lifestyle modifications and treatments in a timely manner, when they are likely to have a greater impact [25,30].

To our knowledge, this is the first study to assess self-reported clinical features of PCOS in a sample of collegiate female athletes compared to non-athletes. Limitations of this study include self-report on surveys, limiting respondents to several states within the northeastern portion of the United States, and a lack of information on the ethnicity of respondents. As a cross-sectional study, it is also unknown if any of the respondents subsequently were or will be diagnosed with PCOS.

## Conclusions

There was an association between being a collegiate athlete and reduced prevalence of hyperandrogenism, menstrual irregularity, and PCOS diagnosis. The athletes in this survey had a lower average BMI and reported more physical activity than non-athletes. Further studies are needed to determine if physical activity, at the level of a collegiate athlete, may help reduce the clinical expression of PCOS diagnostic features.
